# Supplementary material for: Patterns of Intron Gain and Loss in Fungi
Source: PLoS Biol. 2004 Nov 30;2(12):e422. doi: 10.1371/journal.pbio.0020422 (PMC532390; doi:10.1371/journal.pbio.0020422)
Supplement: Table S1 — Also available at http://genes.mit.edu/NielsenEtAl/. (4.3 MB ZIP). [file pbio.0020422.st001.zip › NielsenEtAl/html/1446.html]

AN4655.1.NCU05778.1.MG01170.1.FG02624.1


```
 CLUSTAL W (1.82) Multiple Sequence Alignments - Introns Inserted


Sequence 1: NCU05778.1	185 aa
Sequence 2: FG02624.1	211 aa
Sequence 3: MG01170.1	122 aa
Sequence 4: AN4655.1	173 aa
Alignment Length: 212 aa
Number Identitical Residues: 62 aa
Alignment Score (without introns) 3275


MG01170.1 	---------------------------------------------------~--------
NCU05778.1	MFSRSVRAVSGR--VLANAVRPTVMPAARQIAPAVAV-VAKRSYHEKVLDH1YSRPRNVG
FG02624.1 	MFARGIRAVSRRAAIAAPLARPTILPATRQIAPAVAV-NSSRSYHEKVLDH~YSRPRNVG
AN4655.1  	----------------MSSPLPWSGPTMRKIRSSPNLGYGLKANLLKLIDH~YSNPRNVG
          	                     .   .:  .  .:   . .  :   .  .   : . . .

MG01170.1 	-MDRKDPMVGTGLVGAPACGDVMKLQIKVD--NGTITDAKFKTFGCGSAI~ASSSYLTEL
NCU05778.1	TLDKKDKSVGEGLVGAPACGDVMRLHIKVDPETGVISDVKFKTFGCGSAI2ASSSYVTEL
FG02624.1 	SMNKKDADVGTGLVGAPACGDVMKLQIRVDPETQKISEVKFKTFGCGSAI~ASSSYLTEL
AN4655.1  	SLNRKSLDVGTGLVGAPACGDVIRLDIQVDEATGKITETRFKTFGCGSAI~ASSSYLTTL
          	::::*.  ** ***********::*.*:**  .  *::.:********** *****:* *

MG01170.1 	VKGMRI~EDAAKIRNTDIANNL~HLPPVK-------------------L1HCSMLAEDAI
NCU05778.1	VRGMTL~EQASKVKNTEIAKEL0CLPPVK-------------------L~HCSMLAEDAI
FG02624.1 	VRGMSL~DDAGKVKNTEIAKEL~CLPPVKRTLSHHAAYYIDTQTDFNIV~HCSMLAEDAI
AN4655.1  	LKGKTL1EEASKISNTQIASEL~CLPPVK-------------------L1HCSLLAEDAV
          	::*  : ::*.*: **:**.:*  *****                   : ***:*****:

MG01170.1 	RAAIADYHTKNP---PTNLGKSEVKTLEMEK------
NCU05778.1	KAAIKDYRTKNPHAMPTNLAGTGAKFETAQAA-----
FG02624.1 	KSAISDYYTKNPNAKVSNLSGTGMKLPEADAPAAATA
AN4655.1  	TAAIKNYRSKRAAP-ATDLSGTAKEIPKEAAATA---
          	 :** :* :*.. .  ::*. :  :      .::
```
